# Growth of *Heterostegina depressa* under natural and laboratory conditions

**DOI:** 10.1016/j.marmicro.2015.11.005

**Published:** 2015-11-15

**Authors:** Wolfgang Eder, Antonino Briguglio, Johann Hohenegger

**Affiliations:** a University of Vienna, Department of Palaeontology, Althanstrasse 14, 1090 Vienna, Austria; b Universiti Brunei Darussalam, Faculty of Science, Jalan Tungku, BE1410, Brunei Darussalam

**Keywords:** Larger benthic foraminifera, Tomography, Cyclicity Biometry, Biological oscillators

## Abstract

The use of micro-computed tomography (μCT) provides a unique opportunity to look inside the shells of larger benthic foraminifera to investigate their structure by measuring linear and volumetric parameters. For this study, gamonts/schizonts and agamonts of the species *Heterostegina depressa* d'Orbigny were examined by μCT; each single chamber's volume was digitally measured. This approach enables cell growth to be recognised in terms of chamber volume sequence, which progressively increases until reproduction occurs. This sequence represents the ontogeny of the foraminiferal cell and has been used here to investigate controlling factors potentially affecting the process of chamber formation. This is manifested as instantaneous or periodic deviations of the realised chamber volumes derived from modelled growth functions. The results obtained on naturally grown specimens show oscillations in chamber volumes which can be modelled by sums of sinusoidal functions. A set of functions with similar periods in all investigated specimens points to lunar and tidal cycles.

To determine whether such cyclic signals are genuine and not the effects of a theoretical model, the same analysis was conducted on specimens held in a closed laboratory facility, as they should not be affected by natural environmental effects. Surprisingly, similar cyclicities were observed in such samples. However, a solely genetic origin of these cycles couldn't be verified either. Therefore, detailed analysis on the phase equality of these growth oscillations have been done. This approach is pivotal for proving that the oscillatory patterns discovered in LBF are indeed genuine signals, and on how chamber growth might be influenced by tidal currents or lunar months.

## 1 Introduction

Larger benthic foraminifera (LBF) are an informal group of benthic, symbiont-bearing, marine shallow-water foraminifera that commonly possess a volume larger than 3 mm^3^ (Ross, 1974). They host phototrophic algal symbionts within their shells, thus functioning as greenhouses (Lee and Hallock, 1987; Lee, 2006; Hohenegger, 2011b). The need to provide their symbionts with sufficient light, restricts LBF to the photic zone, forcing LBF to build shells in equilibrium with the physical constraints of their environment such as hydrodynamic energy, light penetrations or nutrient influx (Hohenegger, 2004; Briguglio and Hohenegger, 2009, 2011). The complexity, beauty and giant size of these tests has long attracted scientific interest and revealed interesting data on the biology and ontogeny of these protists (e.g., Lee et al., 1979; Hottinger, 1982; Hallock, 1985; Beavington-Penney and Racey, 2004; Ferràndez-Cañadell et al., 2014). Several studies on the functional morphology and external ornamentation of the shells yielded important information on their ecological niches and distribution (Renema and Troelstra, 2001) in terms of water depth (Hottinger, 2006b), trophic resources (Hallock, 1988) and light intensity (Hohenegger, 2009). According to Hohenegger (2004), primary ecological factors correlate in a non-linear or discontinuous way to water depth. Temperature is an important factor controlling the distribution of most LBF; the critical temperature of their habitat should never fall below 14 °C. In tropical and subtropical regions, the water depth characterising this temperature limit is much deeper than the depth limit based on light.

Light intensity plays a very important role in influencing the water depth distribution of larger benthic foraminifera. Accordingly, the different species occupy various niches along the light gradient (Hohenegger, 2000). In *Heterostegina depressa*, light is noted to be an inverse restriction, since it copes better with low light conditions than with high light conditions (Nobes et al., 2008). Since light intensity changes not only with depth (different penetration of wavelengths) but is also influenced by different factors of water quality (e.g., content of inorganic and organic particles, heightened turbidity, submarine topography), a general correlation between water depth and species distribution is difficult to approach (Hallock et al., 2003; Hohenegger, 2004). Uthicke and Nobes (2008) showed that not all symbiontic larger foraminifera are equally influenced by a change in water quality. For *H. depressa* no distributional changes in accordance with water quality could be found by the authors. Additionally, they emphasise the connection between attenuation of light and lower depth limit of foraminifera.

Wind-induced hydrodynamic motion is one major factor, which can be correlated directly to water depth because it decreases with depth. This dependency, however, varies due to changing wind intensities and the presence of sublittoral and/or tidal currents. For unidirectional hydrodynamics (e.g., tidal and ocean currents) this depth correlation can be further altered by local topography and sea bottom roughness (Hohenegger, 2004).

Apart from that, internal waves can periodically alter temperature and nutrient conditions of meso- and oligophotic biotas (Hallock and Pomar, 2008). Generally, internal waves can be observed along discontinuities within the water columns (e.g., thermoclines and pycnoclines). For shallow water environments surface tides and storms might start internal waves at bathymetric breaks. However while surface currents influence shallow water environments on a larger scale, the influence of internal waves can be restricted to smaller areas (Pomar et al., 2011). Thus, LBF communities can regionally differ at the same water depth due to different energetic conditions and regionally altered water composition. This can be closely observed, when looking at different localities of the Indo-Pacific and Indo-Malayan communities (Ekman, 1953). By looking at west Pacific carbonatic and oligotrophic environments, like Okinawa and Belau, quite similar distributional patterns can be observed. However, for Hawaii, which is a more marginal Indo-Pacific site, a lack of the shallowest subtidal community has been documented (Hallock, 1984). In Okinawa and Belau those are normally dominated by calcarinid taxa. Yet, Hawaii can be seen as a subset of the Indo-Pacific larger benthic foraminiferal community (Hallock, 1984; Hohenegger, 2000).

In comparison communities of the Indo-Malayan regions, like on the Spermonde Archipelago, show also similar distribution, albeit with lower diversity and shallower water depth limits. This is due to higher runoff and higher light attenuation in the mesotrophic mixed siliciclastic environments of the archipelago (Renema and Troelstra, 2001).

Apart from the earlier mentioned factors influencing distribution of larger benthic foraminifera, also seasonal ecological stability (e.g., salinity, influx, nutrients) should be considered as an important factor (Hallock, 1984; Hohenegger, 2000; Renema and Troelstra, 2001).

As the substrate inhabited by LBF is affected by water energy, differences in sediment conditions – firm and soft substrates in combination with the complex interaction of all the factors above – require these organisms to diversify their life strategies. This is reflected in their test morphologies: During the construction of their shell, LBF are strongly influenced by their surroundings, and are forced to reach an equilibrium between their internal physiological need (e.g., growth) and abiotic and biotic external factors. This is reflected within each growth step (i.e., each chamber) of their life (Hohenegger, 2004). Researchers have therefore focused on the chamber-building process and recorded calcification time and symbionts' movement (Spindler and Röttger, 1973), observed calcification potential under different geochemical conditions in relation to climatic variation (Fujita et al., 2011; Hosono et al., 2012) and even confirmed strong pH variation during the chamber-building process (De Nooijer et al., 2009). All this information reveals that the calcification of a new chamber is a complex event that occurs only if many parameters are simultaneously conducive for calcification. This should include also a positive net rate of symbiotic photosynthesis and carbonate availability. However, the exact timing of the chamber-building process is still currently under research and the correlation between chamber formation and environmental conditions is still unknown. Most of the current research deals on how the foraminiferal growth differs along with environmental changes (Prazeres et al., 2015, among others) instead of looking how they normally grow. It is known from cultivation experiments on *H. depressa* that megalospheric specimens apparently follow a quite strict pattern of chamber-building events (Röttger, 1972), therefore suggesting weak correlation with environmental variations.

Chamber growth is intrinsically controlled by genetic factors, but constantly or abruptly changing environmental conditions might influence this process. However the exact trigger of foraminiferal biomineralisation events is so far unknown. Hence, the degree of morphogenetic variability can be higher than caused by the genetic programme and could vary among taxa. Some taxa may have very strict “morphogenetic algorithms”, while others are more susceptible to environmental factors (Tyszka, 2004). Accordingly, changes in chamber size and shape during the chamber-building process might serve as information sources to investigate environmental conditions in the past, using living LBF as control fauna.

The present study concentrates on chamber size, represented by volume measurements, using micro-computed tomography (μCT). This technique enables estimating the volume of each chamber within single tests, revealing the ontogeny of the cell.

Recently, the sequence of chamber volumes has been reported to oscillate around theoretical growth functions. These oscillations have been shown to correlate with tidal, lunar and environmental signals (Briguglio and Hohenegger, 2014; Hohenegger and Briguglio, 2014); to test whether such oscillations reflect environmental oscillations, the same study has been conducted on specimens naturally grown and cultivated under laboratory conditions. The stable culture conditions should inhibit any environmentally induced oscillatory growth. In addition, the comparison between two Indo-Pacific localities (Okinawa and Hawaii) will test how strong geographical and seasonal differences are reflected in the growth oscillations of *H. depressa*.

## 2 Materials and methods

**Fig. 1:**
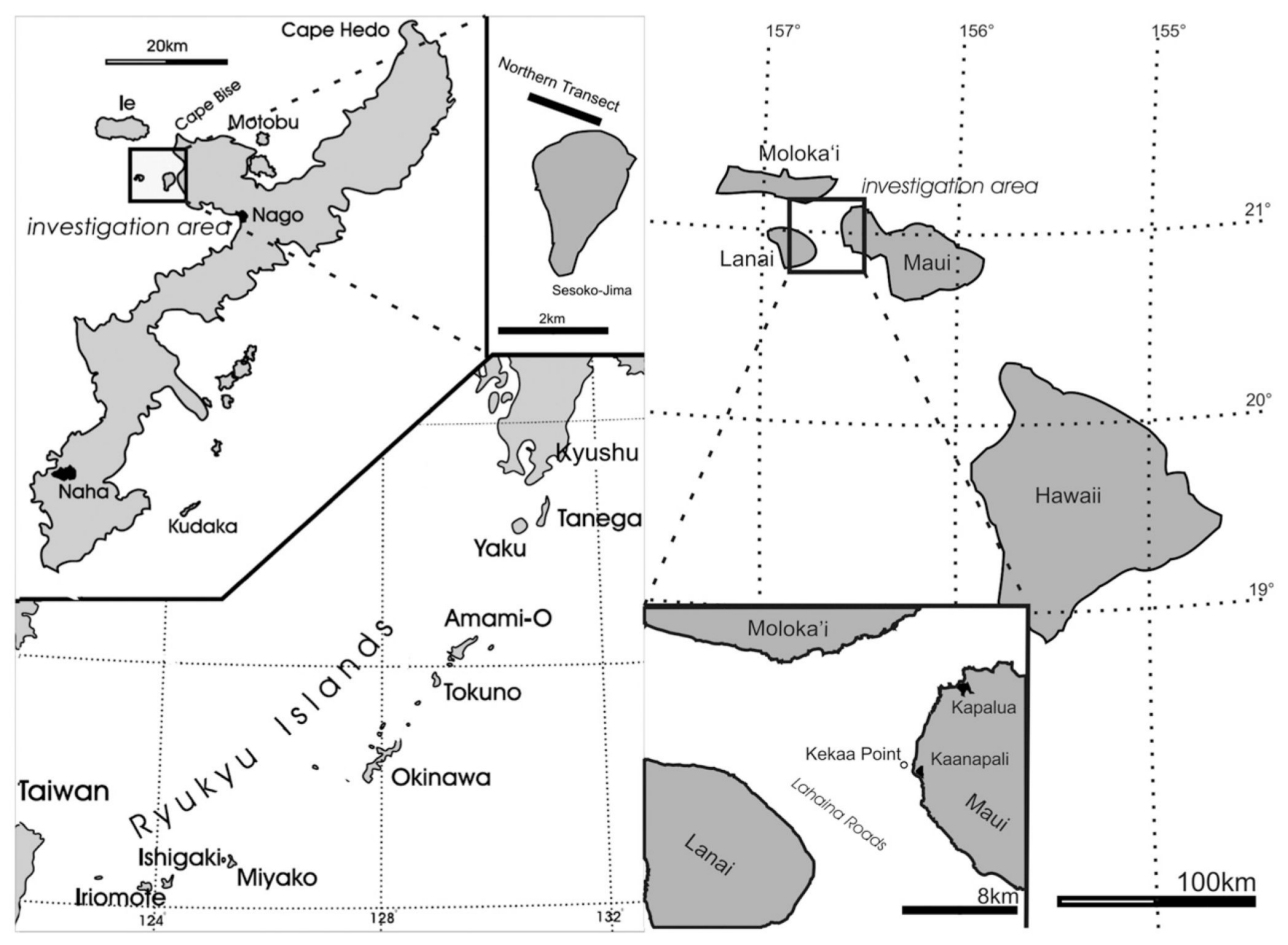
Maps of the sample localities: a. Sample locality Sesoko-Jima: Okinawa, showing transect A and B; after Hohenegger et al. (1999). B. Sketch of western Maui coastline: sampling area of Krüger (1994) accented.

The species selected for these analyses is *H. depressa* d'Orbigny: it constructs chambers divided into chamberlets, which are arranged in a coil that can be approximated by a modified logarithmic spiral (Hohenegger, 2011a; Fig. 3). This shell structure and its biological implications are broadly discussed in the literature (Spindler and Röttger, 1973; Röttger et al., 1984; Hottinger, 2000; Briguglio et al., 2011; Hohenegger, 2011a, 2011b). Additionally, Röttger (1972) published growth data reporting the chamber-building rate for a time span of one year (see Figs. 1 and 4 in Röttger, 1972). This data set has been used to estimate the lifetime and growth pattern of this species using growth functions (i.e., the Michaelis–Menten function) (Hohenegger and Briguglio, 2014), which have been used to estimate the environmental cycles in the analyses presented here. In fully grown individuals, the average chamber number of 60 for gamonts/schizonts (all investigations have been done on empty tests, therefore the megalospheric tests are called gamonts/schizonts) and 100 chambers for agamonts is sufficient to detect cycles.

Fifteen specimens were used in this work (see Table 1, in Supplementary data). The samples consist of six naturally grown gamonts/schizonts collected from Maui, Hawaii (D1-68, D2-68, D3-68), and Sesoko-Jima, Japan (A1, A2, A3), one naturally grown agamont from Sesoko-Jima (B1) and four naturally grown agamonts from Hawaii (B13, B30, B44, B69) as well as four gamonts/schizonts cultivated by Röttger in 1991 at the University of Kiel, Germany (R1, R2, R3, R6).

The Hawaiian specimens used here, belong to the private collection of Röttger and Krüger. The samples originating from Maui, Kekaa Point (20° 55′ 38.38″ N, 156° 41′ 55.91″ E, 20.7.–25.7.1991; Fig. 1) were dredged between 15 to 60 m water depth and split into 0.8, 2.8 and 5.0 mm fractions. The gamonts/schizonts used in this study originate from the 2.8 mm fraction, which were collected at 40 m water depth. They originate from a living and “fresh”-dead assemblage (Röttger, pers. comm.). The “fresh”-dead specimens might have originated from an earlier reproduction season or were transported from other locations. Only the fraction above 5 mm in size was searched for living agamonts, which is where the investigated gamonts come from. The exact water depth of those specimens is unknown (Krüger, 1994). According to Krüger (1994), 113 agamonts were sampled at Kekaa Point and maintained at 22 °C in clear “open ocean water”. Afterwards, they were shipped to the University of Kiel and maintained at 25 °C, 450 Lux at a day–night interval of 12/12 h. Half synthetic seawater was used as a culture medium; the mixture was based on Helgolandian seawater (northern Germany) with 30 to 33‰ salinity and was enriched with concentrated “simple synthetic seawater” (sensu Hauenschild, 1962), enhancing the salinity to 35‰ (Krüger, 1994). The gamonts/schizonts R1, R2, R3 and R6 originated after 76 days of captivity (Krüger, 1994) from one of those agamonts kept in culture from 12.08.1991 until their reproduction on 27.10.1991.

The specimens A1, A2, A3 and B1 were collected at Sesoko-Jima (26° 39′ 38.776″ N, 127° 51′ 56.28″ E, 1.6.–31.7.1996, Fig. 1) around 20 m water depth by SCUBA at transect A described in Hohenegger et al. (1999).

Micro-computed-tomography (μCT), recently applied to observe, quantify and study foraminiferal shells (e.g., Speijer et al., 2008; Briguglio et al., 2011; Görög et al., 2012; Briguglio et al., 2013; Schmidt et al., 2013), was used to more closely examine the internal structure of *H. depressa* by measuring volumes of the chamber sequences within each individual.

**Fig. 2:**
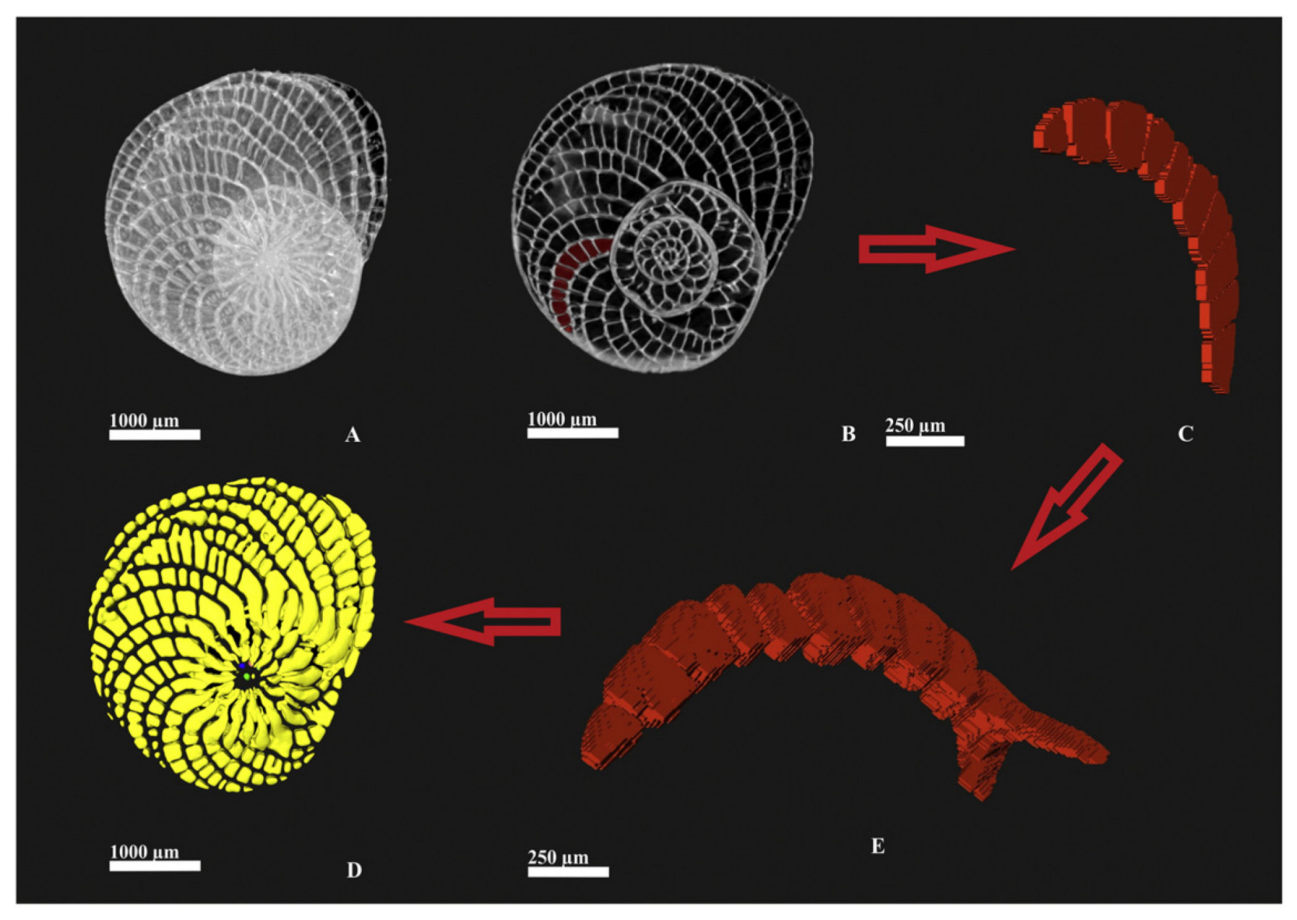
Closer examination of the segmentation process: A: test of specimen D1-68, B: equatorial tomographic slice, C: unrendered model of a chamber, E: single layer of voxels, D: reconstruction of the chamber volume sequence.

Images were taken with the high-energy scanner Skyscan 1173 at the Department of Palaeontology of the University of Vienna (see Briguglio et al., 2014, Fig. 4.1). The dedicated software Amira 5.4.3 VSG was used to work on the three-dimensional models obtained, see Fig. 2.

The most complete specimens of *H. depressa* were chosen as they yield the highest amount of chambers.

### 2.1 Analysis

**Fig. 3:**
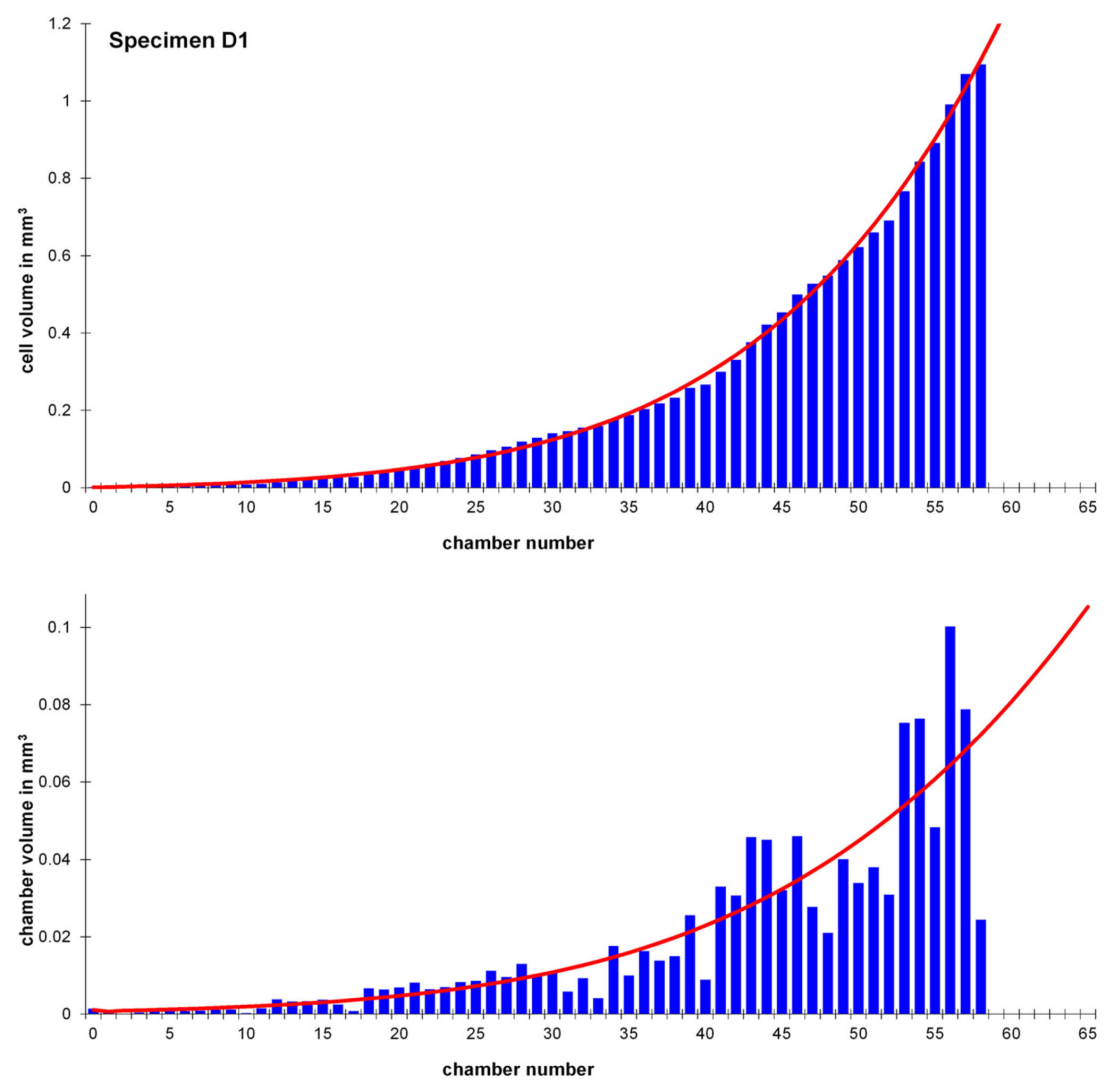
Comparison of the measured cell volumes: The observed cell volume (blue) of specimen D1 (gamont/schizont — Kekaa Point) against the estimated cell volume (red), and of the measured chamber volume (blue) against the estimated chamber volume (red). The oscillations of the measured values around the theoretical growth is visible.

Chamber lumina (sensu Hottinger, 2006a) of each specimen were extracted from the three-dimensional model and their volume calculated; these were summed up to obtain a cumulative distribution representing the overall cell growth (Fig. 3).

This dataset can be fitted by different functions explaining limited growth (see Hohenegger et al., 2014, Fig. 3). The generalised logistic function (Eq. (1), Richards, 1959) allows the best modelling of growth in naturally grown specimens.

(1) $$V_{e}=A+(K-A)/{\left(1+Qe^{-B(j-M)}\right)}^{1/v}$$

The six parameters *A* (lower asymptote), *K* (upper asymptote), *Q* (relation to *Ve*(0)), *B* (growth rate), *M* (represents the starting time *t*_0_) and *v* (position of maximal growth) were estimated using SPSS statistics v. 18.0 (see Table 2 in the Supplementary data). Additionally, an exponential fit for the initial chambers of the gamonts/schizonts (e.g., up to the first 25 chambers) allows a better comparison of the individual cell growth, because of strong growth deviations in later chambers. This aberrance is shown as an increasing fluctuation in later chamber volumes. Therefore, the datasets include for most specimens the chambers of the first to second spiral and represent the growth before the full onset of the “maturo-evolute” growth stage (sensu Banner and Hodgkinson, 1991). This is done using the equation

(2) $$V_{e}=a\;e^{bj}.$$

The parameters *a* and *b* of the exponential function were estimated using SPSS 18 (see Table 2). Additionally, a one-way ANOVA combined with a post-hoc test was done on the above parameters also using SPSS 18.0.

Afterwards the first derivatives of the *V_ e_* values, gained by the generalised logistic function, for each chamber were computed to compare these to the observed chamber volumes, as seen in Fig. 3.

To quantify differences between the observed volume and the theoretical (expected) ones, standardised residuals of the chamber volumes were obtained using Eq. (3)

(3) $$d_{j}=\frac{v_{oj}-v_{ej}}{v_{ej}}$$

where *v_oj_* represents the measured (observed) chamber volumes and *v_ej_* the first derivative of the generalised logistic function for the *j^th^* chamber. Residuals depict how intensively the predicted data of the regression model deviate from the measured data and may represent periodic or instantaneous deviations from the estimated growth function.

To obtain time-dependent periodic functions, this dataset has to be related to the chamber-building rate in order to reveal oscillations and cyclic patterns related to time in days.

**Fig. 4:**
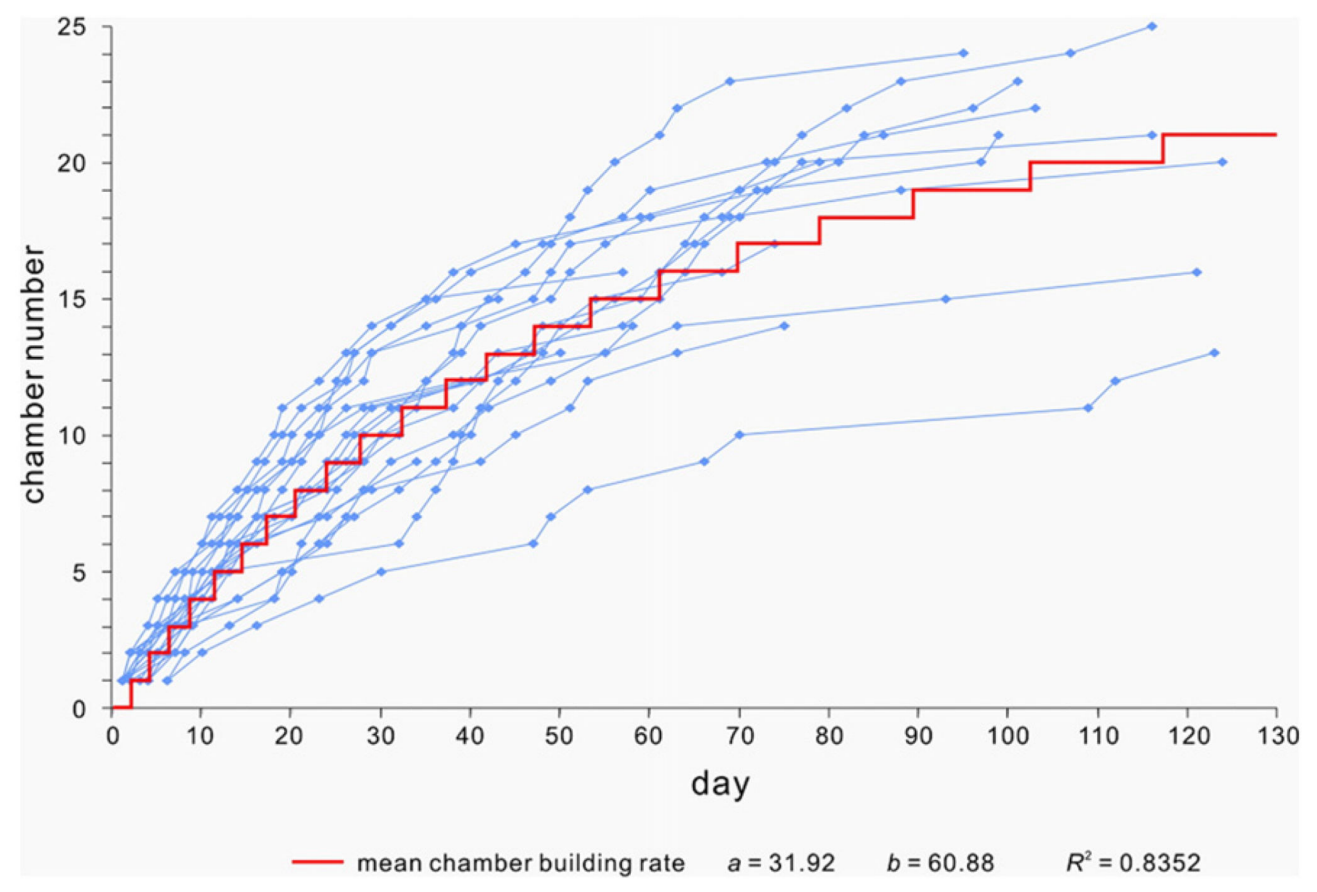
Chamber building rate of 20 *H. depressa* specimens from laboratory cultivation and fit by Michaelis–Menten function (from Hohenegger and Briguglio, 2014).

The chamber-building rate is based on laboratory observations of *H. depressa* (Röttger, 1972) and can be approximated by the power function that poses as a mean chamber building rate. Therefore individual chamber building rates might deviate from the given function (Fig. 4).

However, the used dataset is limited to one experimental setup with specimens originating from a single locality. Therefore, how the difference in population or environmental factors change the timing of chamber-building events cannot be taken into consideration. Based on this assumption, the following equation can be used to express the chamber-building rate

(4) $$j=1.4t^{0.64}$$

where *t* is the time when chamber *j* has been built.

Eq. (4) must be inverted to obtain the timing of chamber formation, resulting in

(5) $$t_{j}={\left(j/1.4\right)}^{1/0.64}.$$

Since no data are available on chamber-building rates for agamonts, a theoretical growth function was estimated based on gamont/schizont data, considering that the chamber-building rate in agamonts should differ from gamonts/schizonts. During the earliest life phases the agamont growth rate should be accelerated, while later life stages show adaptations to a *K*-strategy (Hottinger, 1982, 2000; BouDagher-Fadel, 2008). Power regression was used to approximate to limited functions, like the Michaelis–Menten or Bertalanffy functions, to gain a function for chamber-building rates of *H. depressa* agamonts for a life span of three years. Although the actual agamont lifespan is unknown, it seems to be very close to this value (Hohenegger and Briguglio, 2014):

(6) $$j=4.39t^{0.5}$$

leading to the inverse function for the chamber-building rate in agamonts

(7) $$t_{j}={\left(j/4.39\right)}^{1/0.5}.$$

**Fig. 5:**
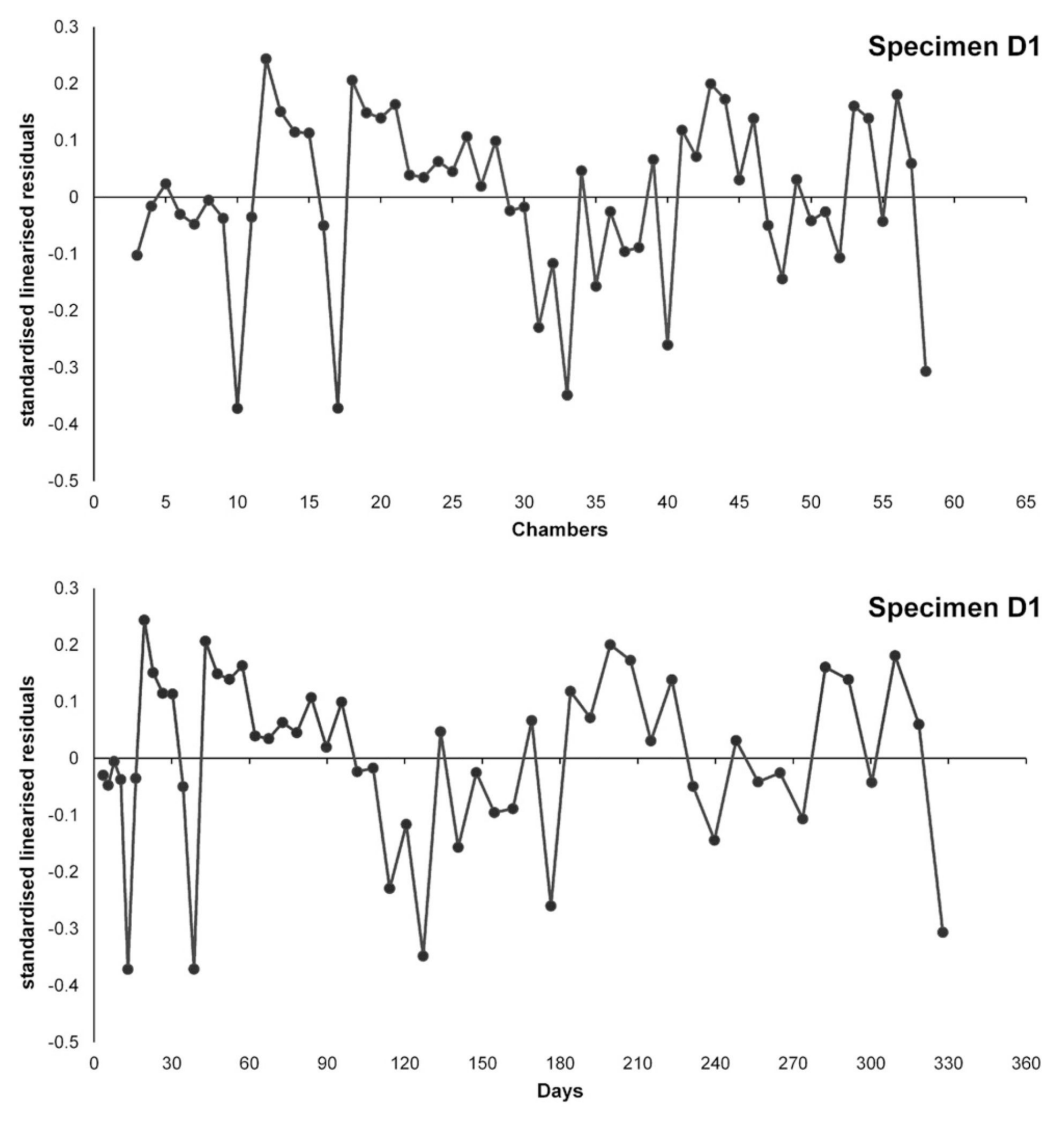
Illustration of standardised and linearised residuals and their transformation from chamber number into days.

For further analyses, residuals were calculated using chamber volumes (Eq. (3)) that are linearised by cubic roots, see Fig. 5.

Then, cyclic patterns were sought by power spectra using Lomb periodograms combined with a sinusoidal regression model (Press et al., 1992) as well as by REDFIT spectral analysis (Schulz and Mudelsee, 2002) to check for significant cycles. An oversampling rate of 4 (by Monte Carlo integration) was used to increase the number of points in REDFIT analysis. Cycles exhibiting power >80% χ^2^ false alarm level lines were considered as significant and included in the model.

The computed sinusoidal functions contain all significant cycles with their amplitudes α, phases ϕ and periods τ. Additionally, probability *p* and the coefficient of determination (*R*^2^) for these summed functions are given in the Supplementary data.

Importantly, the basic target of the used method is to find cycles within a given data set; this implies that cycles can be found within every data set whether they are significant or not (Press et al., 1992; Hammer et al., 2001; Schulz and Mudelsee, 2002). Therefore significant periods were also proven based on their frequency distribution. Because of different life-times expressed in chamber number *n* of specimen *j* and differing amplitude height α_*ij*_ of the *i*th period, the periods τ_*ij*_ must not be used as single measurements giving equal weight to all periods by

(8) $$f(\tau_{ij})=1$$

but should be weighted based on amplitudes *a_ij_* by

(9) $$f(\tau_{ij})=a_{ij}.$$

When the resulting frequency histogram of weighted periods is inhomogeneous, it confirms concentration centres around distinct and thus significant periods. Conversely, a more or less homogeneous distribution with wide ranges argues against significant periods.

Logistic functions, exponential functions and their parameters were calculated in SPSS statistics v. 18.0. REDFIT spectral analysis and sinusoidal functions were computed in PAST 3.04 (Hammer et al., 2001), and Microsoft Office EXCEL 2003 was used for other calculations.

## 3 Results

**Fig. 6:**
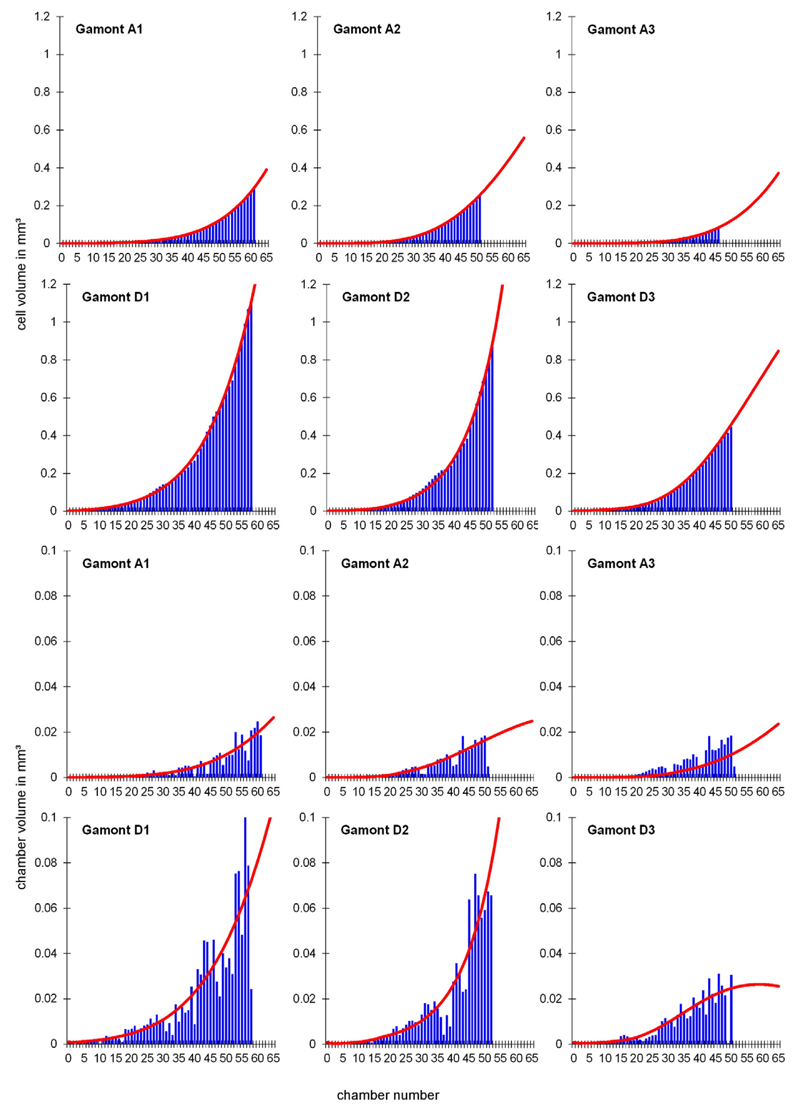
Observed and estimated cell volumes and chamber volumes of all investigated naturally grown gamonts/schizonts (A1–A3: Sesoko-Jima, D1–D3: Kekaa Point).

**Fig. 7:**
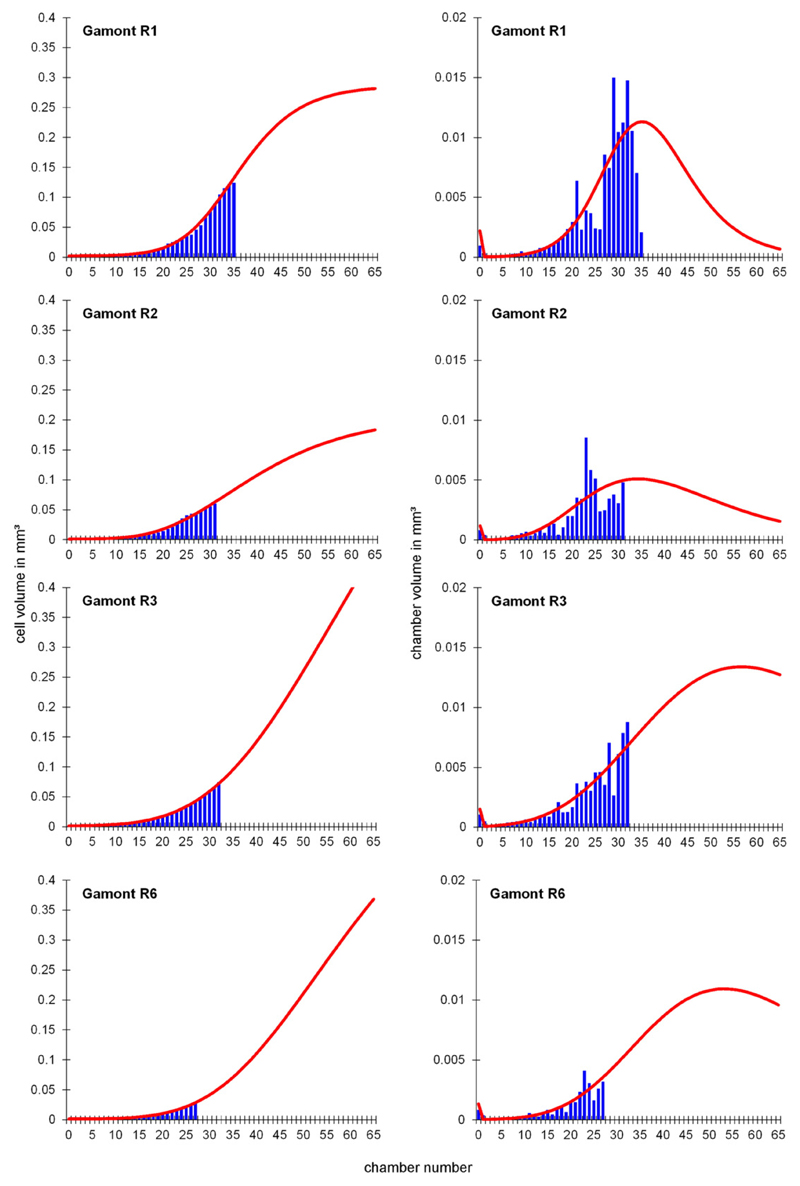
Observed and estimated cell volumes and chamber volumes of all laboratory-cultured gamonts/schizonts.

**Fig. 8:**
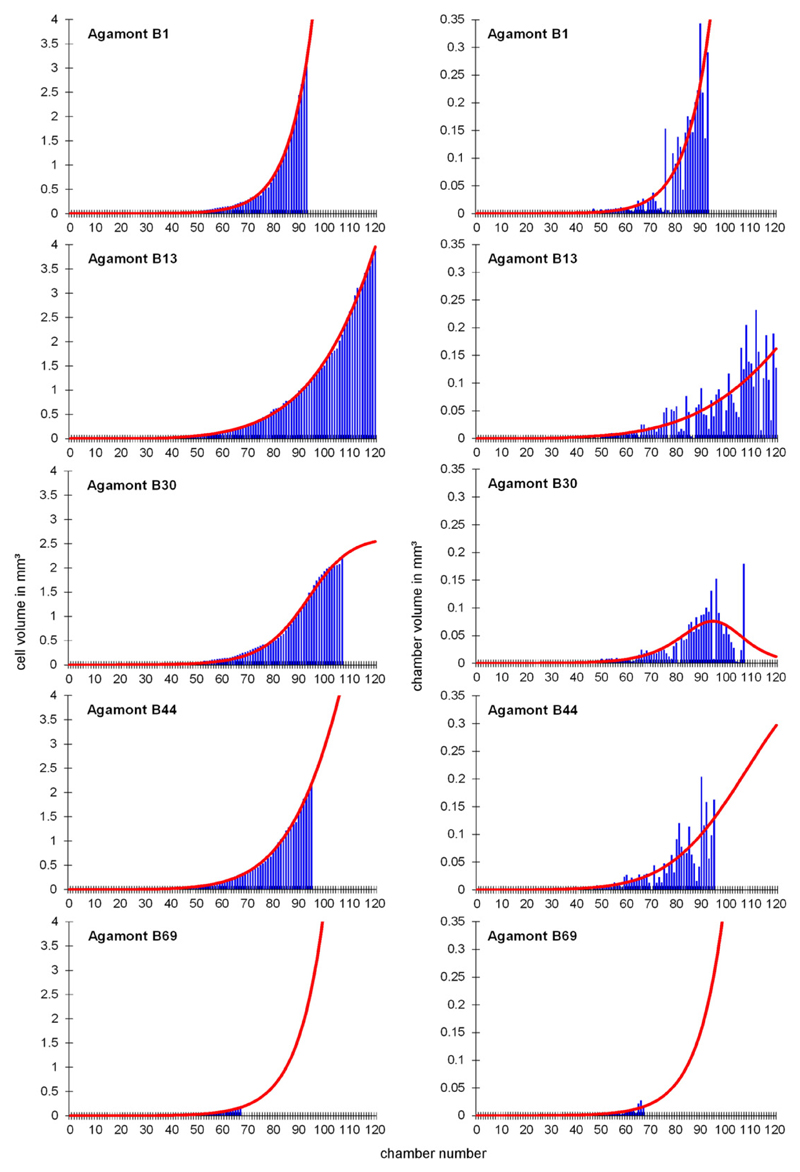
Observed and estimated cell volumes and chamber volumes of all naturally grown agamonts (B1: Sesoko-Jima, B13, B30, B44, B69: Kekaa Point).

**Fig. 9:**
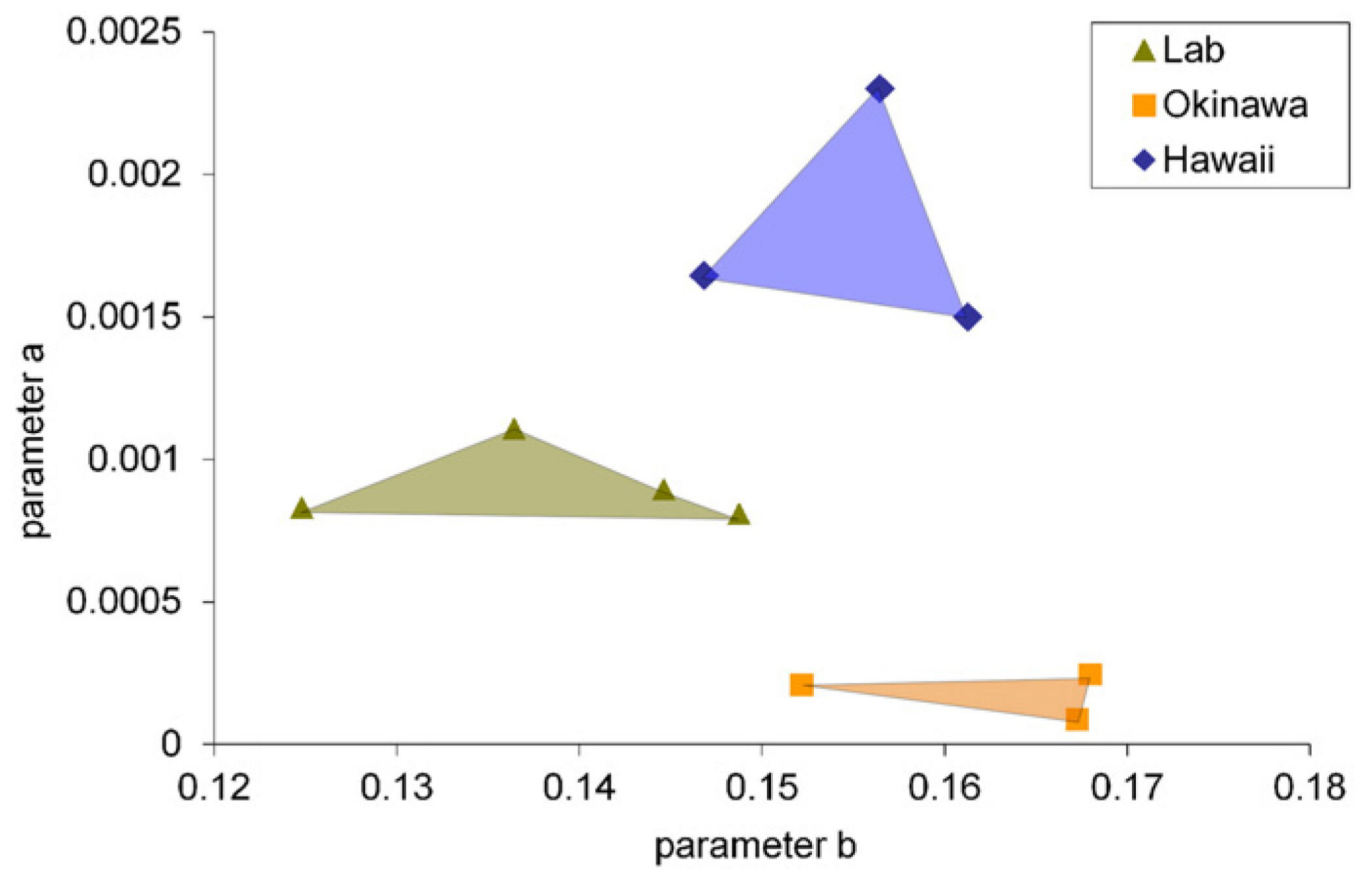
Scatter plot of the parameters for the exponential fit of the first 25 chambers for all investigated gamonts/schizonts.

The most significant periodic functions of each specimen with amplitudes α, phases ϕ and periods τ, as well as *R*^2^ for correlation and probabilities *p* and the parameters for the generalised logistic functions and the exponential functions for the initial spiral are given in the Supplementary data (Tables 1 and 2). These functions describe the foraminiferal cell growth. Figs. 6, 7 and 8 show the observed versus estimated cell and chamber volume of naturally grown gamonts/schizonts, laboratory-cultured gamonts/schizonts and naturally grown agamonts. Fig. 9 shows a scatter plot of the parameters for the exponential fit to initial cell growth of all investigated gamonts/schizonts. These parameters have been used for a one-way ANOVA with a post-hoc test, where the results are given in the Supplementary data.

For the observed cycles in foraminiferal growth, histograms on weighted frequencies are presented for the Hawaiian and Okinawan gamonts/schizonts and the cultivated Kiel specimens, as well as for the agamonts from Kekaa Point and Sesoko.

**Fig. 10:**
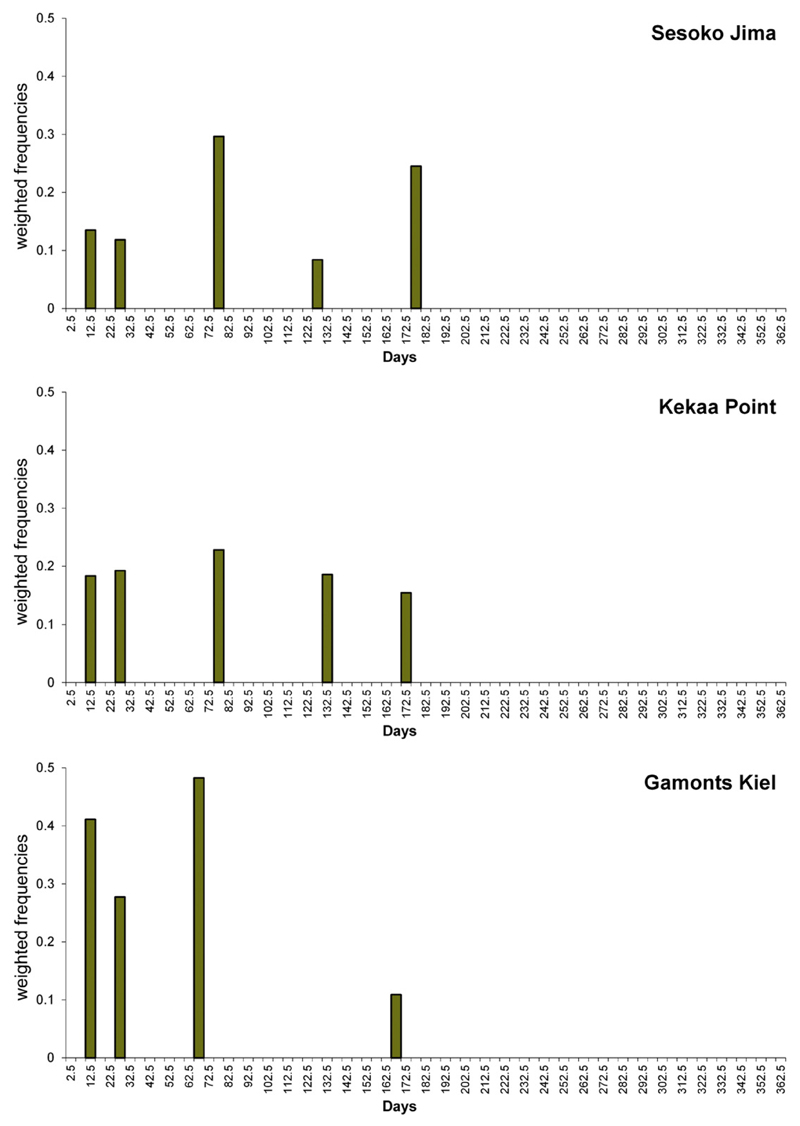
Histograms of significant weighted periods for the naturally grown gamonts/schizonts of Kekaa Point and Sesoko-Jima.

Periods with an average length of 14.75 (SD: 0.03), 28.6 (SD: 0.8), 75.9 (SD: 2.0), 129.8 (only present in one specimen) and 176.3 (SD: 3.2) days were the most significant in naturally grown gamonts/schizonts from Sesoko Jima (Fig. 7). For the Hawaiian gamonts/schizonts, the dominant values were 14.1 (SD: 0.5), 27.8 (SD: 0.9), 76.5 (SD: 3.3), 130.5 (SD: 3.9) and 173.8 (SD: 1.12) days (Fig. 10).

The significant periods in the cultivated gamonts do not differ from those of naturally grown ones on a large scale. These specimens showed cycles with broad ranges at an average period length of 14.8 (SD: 0.4), 27.8 (SD: 0.2) and 69.9 (SD: 4.6), but a single specimen exhibited one period at 165.6 days (Fig. 10).

**Fig. 11:**
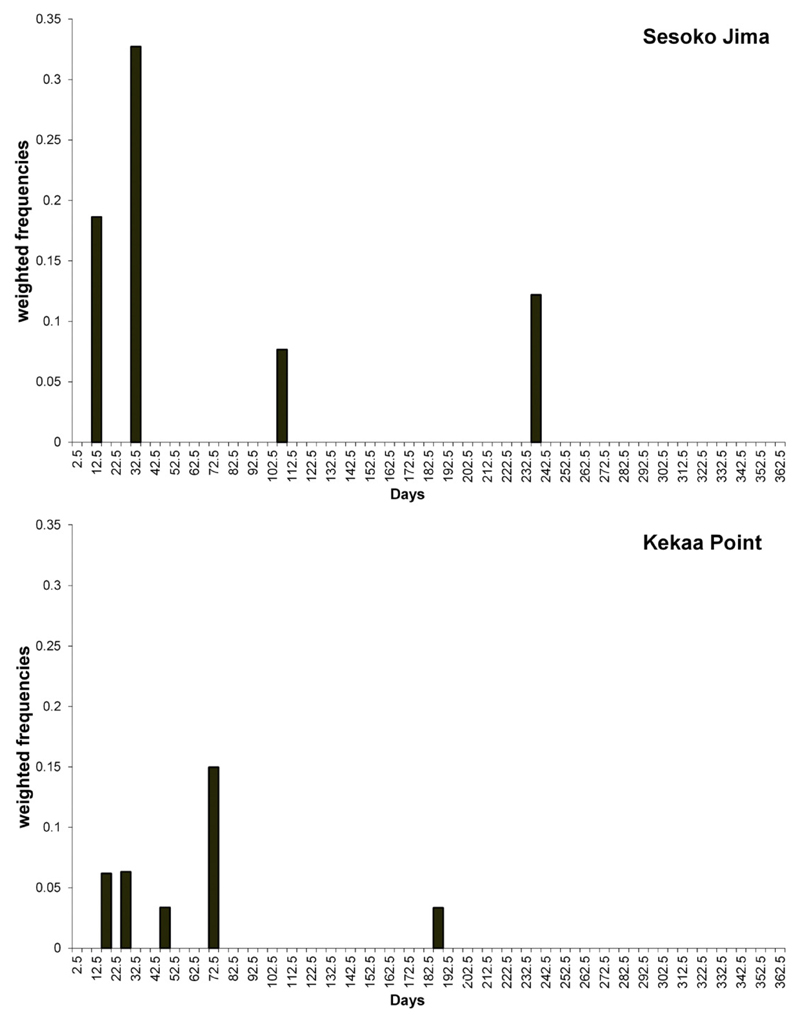
Histogram of weighted significant periods for the naturally grown Sesoko Jima agamont and for Kekaa Point agamonts.

The agamont of Sesoko-Jima showed short-term cycles at 12.7 (SD: 0.1) and 34.6 (SD: 1.7) days and longer significant cycles around 105.9 and 239.5 days. Similar cycles were found in the agamonts of Kekaa Point with the most significant periods around 16.4 (SD: 0.6), 28.5 (SD: 1.2), 47.2 (SD: 0.06), 74.7 (SD: 2.5) and 187.5 days (SD: 0.2) (Fig. 11).

In addition, the μCT investigation revealed that all investigated specimens cultured by Röttger in 1991 showed various internal test anomalies (Hohenegger et al., 2014) such as incomplete septula, undulated septa and the formation of large internal cavities connecting multiple consecutive chambers. Such malformations were not clearly visible from the external surface of these cultivated specimens (Krüger, 1994). Afflicted chambers have been excluded from the analysis.

## 4 Discussion

By measuring and computing chamber volumes and cell volume, the growth of *H. depressa* can be investigated more thoroughly than in two dimensional studies focusing on the foraminiferal growth. The chamber volume represents every growth step of the foraminiferal cell, therefore the chamber volume sequence allows detailed modelling of the cell ontogeny.

Generally speaking, the overall cell growth of *H. depressa* follows a restricted growth model, as predicted by the Gompertz (1825), Michaelis and Menten (1913) or von Bertalanffy function (Bertalanffy, 1951). This major scheme is evident in all investigated specimens. The best fit of these observed chamber volumes is given by the generalised logistic function (Richards, 1959), which results in accurate estimation of both initial cell growth, and the successive life stages. Even though the Richards' curve allows the most precise alignment to natural growth, its complexity and high number of parameters hinders a direct comparison between different individuals. Therefore an exponential fit of the initial spiral (e.g., first 25 chambers, including pro- and deuteroloculus) was used to generate the two comparable and significant parameters *a* and *b*. While *a* represents the initial size (more or less the proloculus volume), *b* represents the individual growth rate. These two parameters were observed to reflect distinct information either on provenance or on ecology. In the investigated specimens the initial size showed a clear dependence on locality, as seen in Fig. 9.

Hawaiian gamonts/schizonts have a much larger initial size than the representatives of the Okinawan population, while the laboratory-cultured gamonts/schizonts, which originate from the Hawaiian population, show an intermediate initial size in-between the natural grown specimens of both localities. This allows two interpretations: either the different proloculus size is a genetic trait of the population and might show an evolutionary trend within the taxon; or the initial size is influenced by an inherent ecological parameter of those geographic localities. However, it is intriguing that laboratory-cultured specimens have a smaller proloculus size than their natural relatives. This might either imply that the initial size depends indeed on an ecological parameter, which couldn't be simulated in the petri-dish, or the reduced embryonic size is due to suboptimal culturing conditions.

The parameter *b* gives the increase of the growth function, meaning the growth rate. It is apparently much more similar in natural grown specimens of different localities than natural and cultured specimens originating from the same population. Therefore, it is most likely that parameter *b* has a higher ecological plasticity than parameter *a*. This might be an additional indicator for the discrepancy between simulated environments and actual natural conditions as assumed by Hohenegger et al. (2014). The complexity of ecological variables affecting the growth of LBF thus cannot be easily substituted by laboratory conditions and should be always combined with continuous field observations (‘natural laboratory’; Hohenegger et al., 2014).

Additional observations on the chamber volume reveals evident periodic patterns in all investigated specimens of naturally grown *H. depressa.* Periods around 14 and 29 days most frequently showed the highest significance, possibly documenting the influence of tides and lunar months on foraminiferal growth. Dependency on moonlight cycles has already been demonstrated within many marine and terrestrial metazoan groups (Winter and Sammarco, 2010; Mercier et al., 2011) and also within planktonic foraminifera based on population dynamic studies (Bijma et al., 1990; Erez et al., 1991; Bijma et al., 1994; Lončarić et al., 2005). One explanation for this correlation between foraminiferal growth and moon phases (every ~29 days) could be that the endosymbiotic microalgae hosted by the LBF have higher photosynthetic rates during full moon periods.

More complicated and speculative is the correlation between oscillations in new moon spring tides and foraminiferal growth. The semidiurnal tidal regime of Sesoko and Hawaii have a periodicity in spring tides of half a lunar month (~14 days). Tides can produce strong and deep tidal currents, which run along the substrate layer, influencing semi-sessile benthic organisms like LBF (Hohenegger et al., 1999; Zuo et al., 2009). Abundant fine-grained deposits can be suspended, diminishing light intensity but increasing inorganic nutrient availability. This might affect foraminiferal endosymbiont activity.

Apart from this quite regional tidal influence, new results of geophysical studies on the seismicity of rifting zones implicate an impact of gravitational forces on the oceans. A strong correlation between lows in ocean tides (fortnightly cycle: 14.5 days) and heightened volcanic activity at mid-ocean ridges, as well as low-magnitude earthquakes has been postulated (Tolstoy et al., 2002; Tolstoy, 2015). These events could influence sea life on a far larger and global scale.

**Fig. 12:**
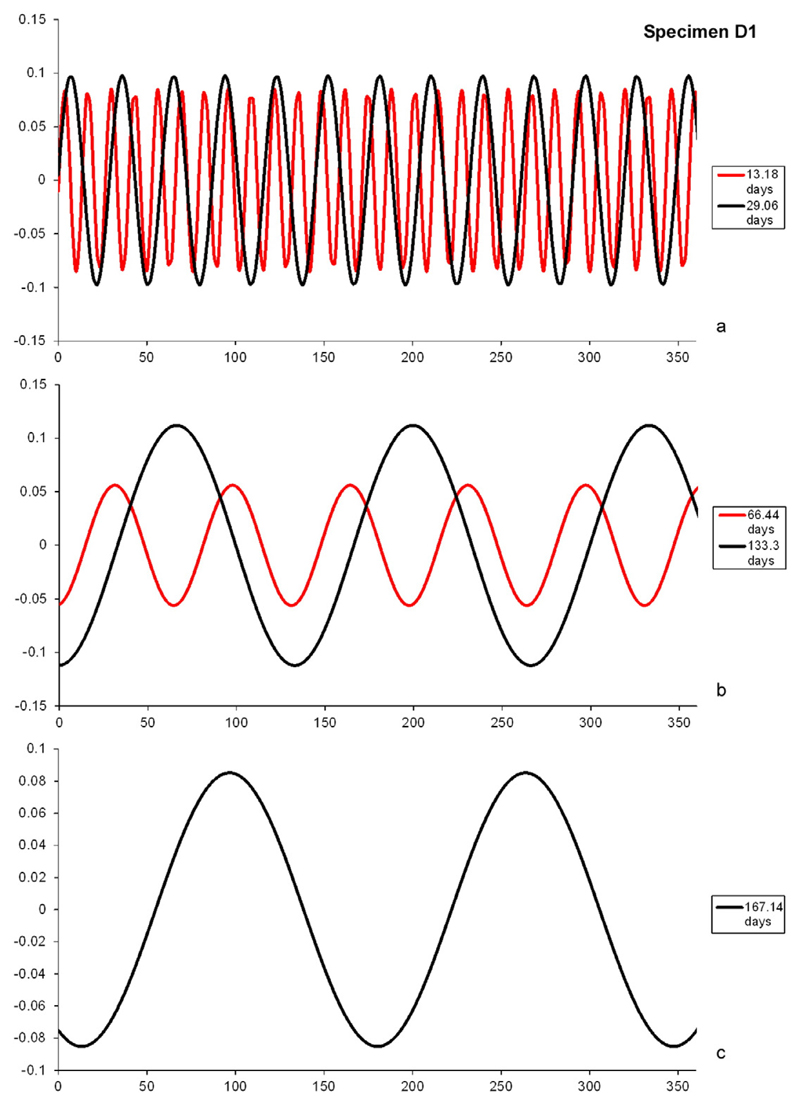
Extracted cycles of specimen D1: a. short-term cycles around 14.5 and 29 days; b. in-phase long-term cycles around 70 and 130 days; c. long-term cycle around 180 days.

**Fig. 13:**
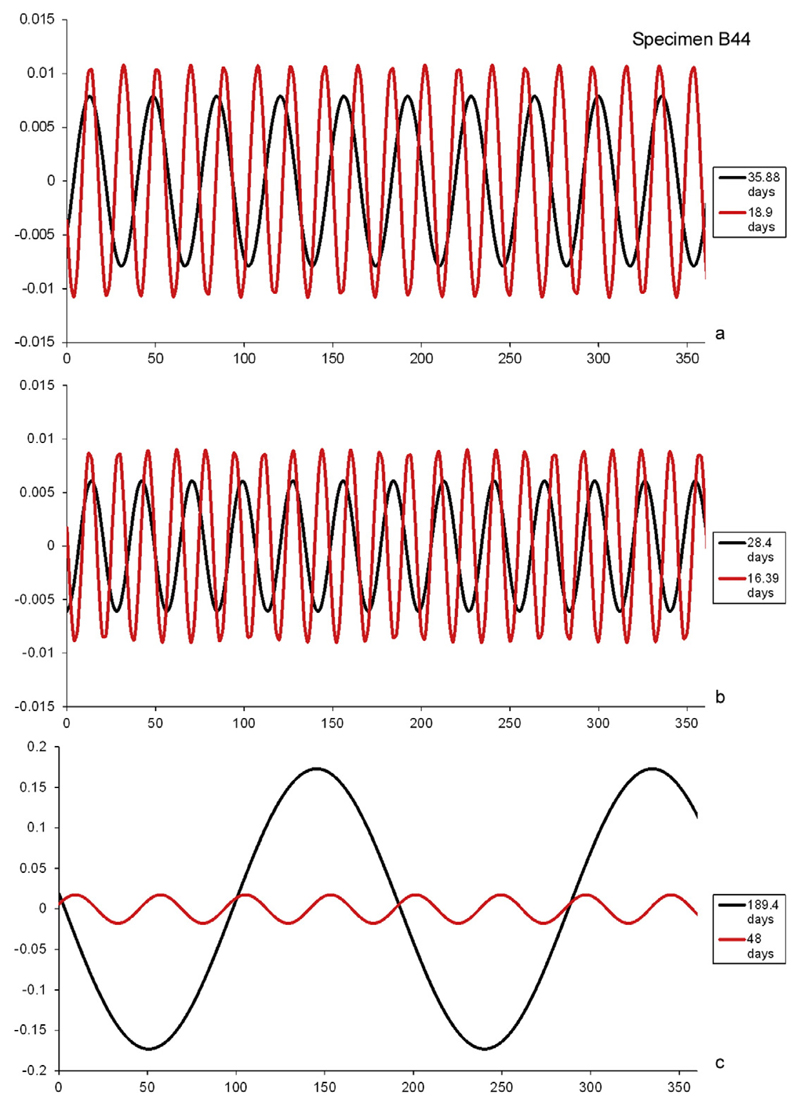
Plot of separately extracted cycles of specimen B44: (a & b) short-term cycles around 14.5 days and 29 days, (c) long-term cycles around 50 days and 180 days.

The cycles observed in LBF growth should be the result of two corresponding effects: light intensity increase due to the moon light and light attenuation due to turbid tidal currents. Therefore, their phases should be always in a correlative context to each other, resulting in a partially constructive or destructive interference (Hohenegger and Briguglio, 2014, Fig. 3.20). This correspondence of cycles around 14 and 29 days could be found in all investigated specimens, gamonts/schizonts and agamonts alike. When plotting those cycles on top of each other, the same pattern of interferences emerges as observed by Hohenegger and Briguglio (2014) (Figs. 12a & 13a, b).

Besides these short-term cycles, some naturally grown specimens also show intermediate periods around 75 and 130 days (Fig. 12b). Agamonts and gamonts/schizonts from both localities exhibit 75 day cycles, while only gamonts/schizonts of both localities exhibit 130 day cycles. The most peculiar feature of these cycles is their corresponding phases and periods, also seen in short-term cycles.

The discrimination between ecological driven cycles and those created by analytical artefacts is hampered by the fact that long-term cycles might be actually the product of an artificial stacking effect of the 14 and 29 day cycles. The same is probably also true for long-term cycles around 170 to 180 days (Figs. 12c and 13c). Although further investigation on different localities with stronger and weaker seasonal changes in salinity, terrigenous influx and nutrients might allow to decipher more accurately, which long-term cycles are genuine. Alas, the environmental and latitudinal similarity between Hawaii and Okinawa (oligotrophic carbonate platforms) might also hinder our ability to see real differences in the periods of long-term cycles. Hence, further analysis from innertropic mesotrophic mixed siliciclastic settings, like Spermonde Archipelago, could result in different long-term cycles. This might be especially interesting for those taxa, that can adapt to a wider range of environmental parameters, like *H. depressa*.

However, one of the most striking results presented here is that, in contrast to expectations, cultured specimens exhibit nearly the same periodic patterns as naturally living individuals.

This allows two possible interpretations: either growth cycles are a general characteristic of foraminiferal growth and are not environmentally controlled, or periodic growth patterns are inherited via epigenetics, but calibrated by ecological rhythmic signals as seen in pulse-coupled oscillators (Bélair, 1986; Mirollo and Strogatz, 1990).

This last interpretation considers that extrinsic rhythms, like lunar or tidal rhythm, might have positive or negative influence on the foraminiferal growth and therefore the organisms adapt and react in equilibrium with their “cyclic” environment. These reactions are afterwards inherited by an environmental maternal effect (Räsänen and Kruuk, 2007; Richards, 2006; Richards et al., 2010) and transmitted to the next generation which still keep the cyclic growth pattern in a non-cyclic environment (e.g., petri-dish). In this way, environmentally induced cycles could become inherent growth cycles.

Furthermore, since individuals from the same locality and reproduction time should exhibit cycles with similar phases, the phase equality within the population should be discussed as well as they have been collected alive or from a living–“fresh” dead assemblage.

**Fig. 14:**
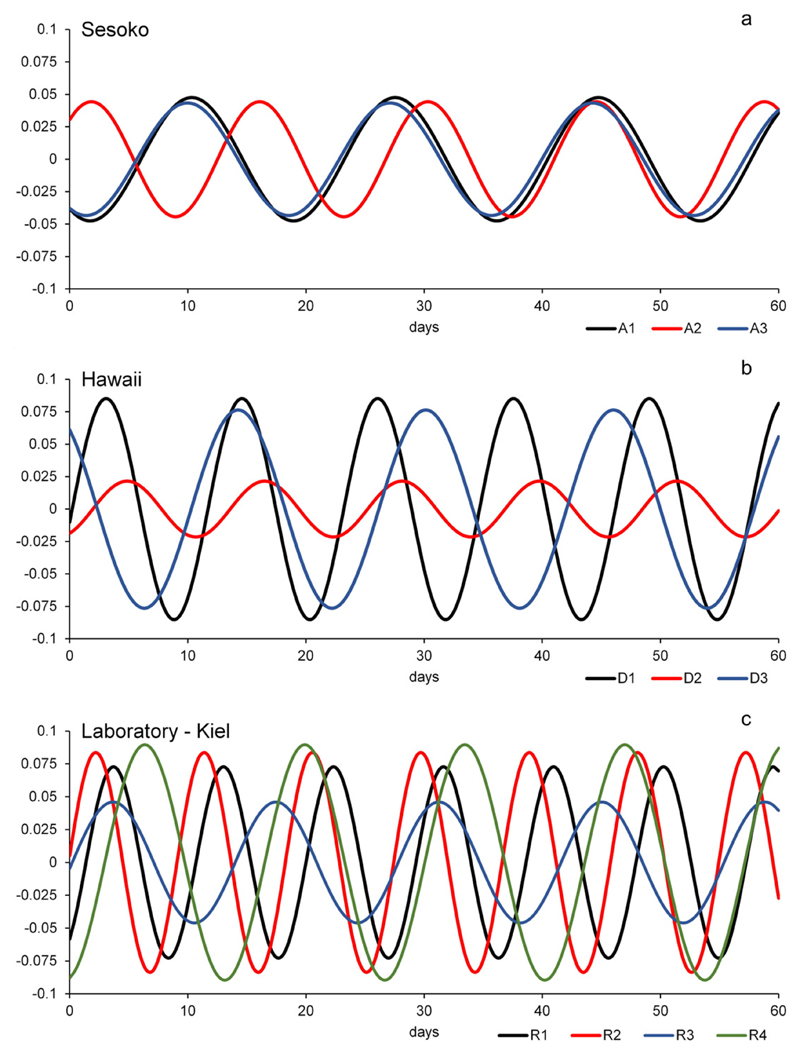
Comparison of the 14 day cycles: The 14 day cycles of all gamonts/schizonts separated by localities to search for phase equality; a. Sesoko-Jima; b. Hawaii; c. lab — Kiel.

In Fig. 14, the extracted 14 day cycles of gamonts/schizonts of each locality are plotted on top of each other to check for phase equality. For the gamonts/schizonts of Sesoko-Jima, which were sampled during the same reproduction time, the phases are either equal or complementary (Fig. 14a). For the gamonts/schizonts of Hawaii, which seem to originate from different reproduction times, phases show a much more randomly scattered pattern than in Sesoko (Fig. 14b). Laboratory-cultured specimens should show aligned phases, since all of them are clones. However this is not the case, the phases seem to be scattered around a common centre (Fig. 14c). Therefore these cycles cannot be plainly intrinsic and probably need an extrinsic pulse to calibrate them, as so-called pulsed-coupled biological oscillators. This mechanism is strongly discussed in biomathematics and it has been thoroughly researched how pulsating signals can influence it. Till now this process has been found in pacemaker neurons, the respiratory rhythm, circadian activity, and in the control of mitosis, but not yet in complex metabolitic activity of protists (Knight, 1972; Sachsenmeier et al., 1972; Buck and Buck, 1976; Petrillo, 1981; Bélair, 1986; Mirollo and Strogatz, 1990). In the given case the aforementioned seismic events during neap tides could pose as the gauging pulsatory signal, which implicates a gravitational-astronomical forcing on foraminiferal ontogeny. However, since this tidal influence on seismicity of rifting zones by Tolstoy (2015) has been discovered quite recently, no research on its influence on Earth's sea life has been done.

Finally, in direct comparison of growth functions in natural grown and laboratory-cultured specimens a clear difference in the mode of cell growth is visible, as seen in Fig. 9, confirming the assumption by Hohenegger et al. (2014) that the complexity of ecological variables affecting the growth of LBF cannot be easily substituted by laboratory conditions and should be always combined with field observations.

## 5 Conclusion

Computed micro-tomography and 3D reconstruction successfully quantifies the ontogeny of foraminiferal cells volumetrically, enabling the volumes of the whole chamber sequence to be accessed. *H. depressa* is a well-studied larger benthic foraminifera and thus represents an excellent model organism for the actuopalaeontological approach used in this work. The results on cell growth via volume analysis imply that not only embryonic size of larger benthic foraminifera give valuable information to reconstruct palaeoecology and biogeography, but also their growth rate might provide new insight in which way their local environment influences cell growth. So far, it can be concluded that embryonic size is a possible indicator to distinguish geographically isolated populations of this nummulitid taxon and maybe also other closely related taxa, as is has been found in other non-nummulitid groups. However, this effect might be also impaired by suboptimal environment conditions during laboratory culture.

The observations on the chamber volume revealed that LBF record, due to their longer lifetime, short- to long-term oscillations during their chamber formation, expressed in chamber size variation. Even though it is most likely that long-term cycles are only mathematical artefacts. Special attention should be given to the chamber-building rates, which are estimated using a power function instead of a Michaelis– Menten or Bertalanffy function, because these have a specific limit for each individual.

The results confirm that naturally grown foraminifera record oscillations in their chamber volume, which can possibly be induced by lunar and tidal cycles. Lunar cycles, and therefore light intensity oscillations, might affect the productivity of the photosynthetic symbionts hosted by the foraminiferal cell, probably causing a positive influence on photosynthetic activity during full moon nights. Certain growth oscillations point to tidal variation, which might reflect the effects of tidal currents (e.g., water turbidity, organic and inorganic nutrient availability) on the cell. Further comparison of specimens from different tidal regimes and/or localities with stronger and weaker seasonal influence might allow a better deciphering of growth cycles, especially long-term cycles. Hence, research on latitudinal changes of long-term cycles has to be carried out in the future to inspect which fluctuating environmental factors influence LBF the most.

The occurrence of similar cyclicities in naturally grown and laboratory-cultured specimens implies that there are much more complex biologic mechanisms influencing these growth cycles. Therefore, a solely environmental cause is implausible and can probably be excluded. Detailed analysis and comparison of phase equality of specimens of each locality showed that the cyclic growth also cannot be only genetically controlled. Hence the theory of pulse-coupled biologic oscillators might apply to the oscillatory growth of LBF. Further and much more detailed research has to be done on cell growth and on growth cycles of these extraordinary protists to reveal the mechanisms of cyclic growth in larger benthic foraminifera.

## Appendix A. Supplementary data

Supplementary data to this article can be found online at http://dx.doi.org/10.1016/j.marmicro.2015.11.005.

Supplementary Data
