# Supplementary material for: Growth of Heterostegina depressa under natural and laboratory conditions
Source: Mar Micropaleontol. Author manuscript; Available in PMC 2017 Jan 16. (PMC5238945; doi:10.1016/j.marmicro.2015.11.005)
Supplement: Supplementary Data [file NIHMS70772-supplement-SupplementaryData.pdf]

Table 1.1

Code	Origin	SampleNo.	Depth (m)	Amplitude						
				α1	α2	α3	α4	α5	α6	α7
**D1-68**	Maui, Kekaa Point	#68	40	0.085	0.098	0.056	0.112	0.085		
**D2-68**	Maui, Kekaa Point	#68	40	0.022	0.039	0.069	0.070			
**D3-68**	Maui, Kekaa Point	#68	40	0.076	0.055	0.103	0.074			
**A1**	Sesoko-Jima	Transect A	20	0.048	0.068	0.128	0.108			
**A2**	Sesoko-Jima	Transect A	20	0.044	0.012	0.062	0.084			
**A3**	Sesoko-Jima	Transect A	20	0.043	0.038	0.106	0.137			
**B13**	Maui, Kekaa Point	P.1.10.91	n.n	0.005	0.007	0.068	0.069			
**B30**	Maui, Kekaa Point	P.1.10.91	n.n	0.014	0.010	0.020	0.014	0.016	0.016	
**B44**	Maui, Kekaa Point	P.1.10.91	n.n	0.009	0.011	0.006	0.008	0.018	0.173	
**B69**	Maui, Kekaa Point	P.1.10.91	n.n	0.004	0.009	0.003	0.006	0.012		
**B1**	Sesoko-Jima	Transect A	20	0.090	0.097	0.071	0.119	0.137	0.077	0.122
**R1**	Universität Kiel	F1.27.1.91	n.n.	0.073	0.061	0.161	0.109			
**R2**	Universität Kiel	F1.27.1.92	n.n.	0.084	0.094	0.182				
**R3**	Universität Kiel	F1.27.1.93	n.n.	0.046	0.046	0.074	0.066	0.044		
**R6**	Universität Kiel	F1.27.1.94	n.n.	0.090	0.057	0.096				

Table 1.2

**Phase**							**Period**							**R²**	**p**
**φ1**	**φ2**	**φ3**	**φ4**	**φ5**	**φ6**	**φ7**	**τ1**	**τ2**	**τ3**	**τ4**	**τ5**	**τ6**	**τ7**		
1.69	1.52	2.96	3.12	-2.65			13.2	29.1	66.4	133.3	167.1			0.666	2.64E-05
2.6300	-2.49	1.490	-3.10				14.3	28.3	60.3	182.1				0.376	1.71E-03
-0.64	-0.61	2.57	3.10				15.2	25.2	92.9	126.4				0.376	7.98E-03
-2.51	2.20	-3.04	-2.55				14.7	28.4	67.3	178.6				0.472	2.09E-05
0.81	0.75	-0.23	-2.11				15.1	29.3	83.4	129.8				0.432	1.27E-03
-2.63	-2.64	-1.65	-2.31				14.5	28.8	82.0	174.6				0.678	4.03E-07
-1.03	1.54	3.11	-0.27				14.3	34.9	74.6	75.0				0.431	2.09E-10
-1.60	-2.09	-2.44	-3.04	2.01	1.33		13.9	16.4	22.3	27.8	46.4	185.5		0,410	9.79E-06
-1.38	-1.92	3.06	2.24	1.22	-1.46		16.4	18.9	28.4	35.9	48.0	189.4		0.460	1.18E-04
-2.90	-1.95	-2.77	2.23	-0.62			11.8	20.8	28.2	34.8	65.1			0.581	4.52E-06
-2.01	0.00	1.73	0.84	2.15	-0.75	2.70	12.1	13.2	24.5	35.2	39.5	105.9	239.5	0.661	3.48E-11
2.50	1.33	0.80	0.77				11.8	28.8	61.8	165.6				0.735	1.09E-05
1.51	-0.54	0.71					10.7	23.4	76.9					0.671	8.52E-05
2.08	1.67	2.32	2.15	1.56			13.4	15.5	21.1	31.8	66.0			0.860	1.09E-05
2.95	-0.69	0.96					16.5	29.6	72.4					0.618	2.42E-03

Table 2

Code	Generalized logistic function						Exponential function	
	A	K	Q	B	M	v	a	b
D1-68	-0.0065	101.89	0.249	0.0201	121.69	0.1439	0.0023	0.156
D2-68	-0.0088	150.52	1.273	0.2075	105.39	2.2504	0.0015	0.161
D3-68	0.0054	1.80	0.800	0.0400	-147.10	0.0002	0.0016	0.147
A1	-0.0004	28.79	1.580	0.0183	-31.45	0.0568	0.0002	0.152
A2	0.0002	10.49	13.546	0.0205	-368.14	0.0006	0.0002	0.168
A3	-0.0013	15.72	2.321	0.0172	-425.64	0.0001	0.0001	0.167
B13	-0.0100	212.99	0.388	0.0117	83.49	0.0574		
B30	0.0154	2.65	0.218	0.1274	107.38	1.3492		
B44	-0.0022	39.84	0.302	0.0290	136.10	0.2428		
B69	-0.0006	83.94	12.126	0.2217	118.41	2.2883		
B1	-9.4370E-08	6.28	9.682	0.0558	5.19	0.1481		
R1	0.0021	0.2866	11.5879	0.1334	10.7629	0.4806	0.0009	0.145
R2	0.00	0.21	1.64	0.07	-114.90	0.00	0.0008	0.149
R3	0.001	0.907	2.088	0.041	-35.632	0.048	0.0011	0.136
R6	0.0013	0.6292	2.1275	0.0492	-14.3389	0.0785	0.0008	0.1248

ONEWAY ANOVA

		Quadratsumme	df	Mittel der Quadrate	F	Signifikanz
parameter_a	Zwischen den Gruppen	,000	2	,000	32,530	,000
	Innerhalb der Gruppen	,000	7	,000		
	Gesamt	,000	9			
parameter_b	Zwischen den Gruppen	,001	2	,001	6,112	,029
	Innerhalb der Gruppen	,001	7	,000		
	Gesamt	,002	9			

POST-HOC TEST

		(I) Group	(J) Group	Mittlere Differenz (I-J)	Standardfehler	Signifikanz	95%-Konfidenzintervall Untergrenze	95%-Konfidenzintervall Obergrenze
parameter_a	LSD	Kiel	Sesoko	,000729756*	,000189996	,006	,00028049	,00117903
			Hawaii	-,000904807*	,000189996	,002	-,00135408	-,00045554
		Sesoko	Kiel	-,000729756*	,000189996	,006	-,00117903	-,00028049
			Hawaii	-,001634563*	,000203114	,000	-,00211485	-,00115427
		Hawaii	Kiel	,000904807*	,000189996	,002	,00045554	,00135408
			Sesoko	,001634563*	,000203114	,000	,00115427	,00211485
	Bonferroni	Kiel	Sesoko	,000729756*	,000189996	,019	,00013553	,00132398
			Hawaii	-,000904807*	,000189996	,006	-,00149903	-,00031058
		Sesoko	Kiel	-,000729756*	,000189996	,019	-,00132398	-,00013553
			Hawaii	-,001634563*	,000203114	,000	-,00226981	-,00099931
		Hawaii	Kiel	,000904807*	,000189996	,006	,00031058	,00149903
			Sesoko	,001634563*	,000203114	,000	,00099931	,00226981
parameter_b	LSD	Kiel	Sesoko	-,023838083*	,007078258	,012	-,04057550	-,00710066
			Hawaii	-,016219199	,007078258	,056	-,03295662	,00051822
		Sesoko	Kiel	,023838083*	,007078258	,012	,00710066	,04057550
			Hawaii	,007618883	,007566976	,348	-,01027417	,02551194
		Hawaii	Kiel	,016219199	,007078258	,056	-,00051822	,03295662
			Sesoko	-,007618883	,007566976	,348	-,02551194	,01027417
	Bonferroni	Kiel	Sesoko	-,023838083*	,007078258	,036	-,04597570	-,00170046
			Hawaii	-,016219199	,007078258	,167	-,03835682	,00591842
		Sesoko	Kiel	,023838083*	,007078258	,036	,00170046	,04597570
			Hawaii	,007618883	,007566976	1,000	-,01604723	,03128500
		Hawaii	Kiel	,016219199	,007078258	,167	-,00591842	,03835682
			Sesoko	-,007618883	,007566976	1,000	-,03128500	,01604723

B-Form	B1	B13	B30	B44	B69
**chamber**	**lin. Vol. mm³**	**lin. Vol. mm3**	**lin. Vol. mm3**	**lin. Vol. mm³**	**lin. Vol. mm³**
10				0,00000658	
11				0,00000983	
12		0,00000421		0,00000983	
13	0,000001333	0,00000518		0,00001255	0,00000316
14	0,000000333	0,00000597	0,00000000	0,00001387	0,00000623
15	0,000000667	0,00001027	0,00000445	0,00001554	0,00000808
16	0,000001333	0,00001185	0,00000794	0,00003213	0,00001387
17	0,000002000	0,00001133	0,00001323	0,00004196	0,00001571
18	0,000004333	0,00001914	0,00001215	0,00004960	0,00001870
19	0,000004333	0,00002256	0,00001889	0,00004855	0,00002511
20	0,000011667	0,00002283	0,00014895	0,00007875	0,00002757
21	0,000010333	0,00004539	0,00010010	0,00008288	0,00003406
22	0,000016667	0,00006593	0,00022330	0,00008498	0,00003494
23	0,000020333	0,00006786	0,00014871	0,00007612	0,00004706
24	0,000030667	0,00005900	0,00011647	0,00012387	0,00006778
25	0,000034667	0,00006321	0,00009890	0,00003951	0,00006066
26	0,000026667	0,00006997	0,00006365	0,00002634	0,00007260
27	0,000019667	0,00006953	0,00010383	0,00008928	0,00009332
28	0,000089667	0,00017997	0,00019900	0,00008472	0,00011176
29	0,000049333	0,00019147	0,00010720	0,00025187	0,00005074
30	0,000070667	0,00016066	0,00016435	0,00028374	0,00008876
31	0,000058667	0,00028181	0,00009782	0,00029858	0,00019762
32	0,000097333	0,00029946	0,00029525	0,00016364	0,00021904
33	0,000128667	0,00045028	0,00032305	0,00007032	0,00019771
34	0,000189667	0,00048180	0,00062371	0,00034432	0,00027049
35	0,000253333	0,00070883	0,00048439	0,00044063	0,00025749
36	0,000212667	0,00069917	0,00067846	0,00064509	0,00043641
37	0,000337333	0,00083305	0,00039114	0,00086448	0,00013406
38	0,000281667	0,00070549	0,00064176	0,00061577	0,00049295
39	0,000286000	0,00075079	0,00059472	0,00064105	0,00033168
40	0,000850000	0,00065220	0,00097191	0,00068539	0,00067679
41	0,000816667	0,00100565	0,00084918	0,00061445	0,00070435
42	0,000785000	0,00086720	0,00126415	0,00082726	0,00044984
43	0,000533667	0,00051103	0,00120748	0,00047030	0,00026952
44	0,001073000	0,00072788	0,00118438	0,00115235	0,00069724
45	0,000955667	0,00065229	0,00099862	0,00102365	0,00039647
46	0,000806333	0,00070698	0,00139060	0,00050032	0,00149957
47	0,000698000	0,00009402	0,00098971	0,00017348	0,00051990
48	0,002987667	0,00075509	0,00067244	0,00054422	0,00077652
49	0,001215667	0,00001405	0,00093774	0,00262646	0,00008165
50	0,001345333	0,00007199	0,00045792	0,00169245	0,00116403
51	0,002503667	0,00184063	0,00277940	0,00270301	0,00081190
52	0,001746333	0,00115647	0,00189641	0,00179428	0,00120310
53	0,002639667	0,00270652	0,00277940	0,00306024	0,00192228
54	0,002909333	0,00241645	0,00277880	0,00287877	0,00151203
55	0,002768333	0,00321720	0,00297130	0,00177646	0,00135427
56	0,002816667	0,00265121	0,00249149	0,00443795	0,00209313
57	0,003267333	0,00321316	0,00323564	0,00363562	0,00191684
57	0,003196000	0,00218925	0,00175215	0,00269090	0,00145839
58	0,003731667	0,00364869	0,00249149	0,00165470	0,00077028
59	0,002958000	0,00357978	0,00264152	0,00795769	0,00117887
60	0,003355000	0,00291493	0,00325440	0,00897923	0,00319351
61	0,002716000	0,00405420	0,00183144	0,00419609	0,00271056
62	0,001858333	0,00323563	0,00109342	0,00718513	0,00354748

63	0,001346667	0,00325214	0,00158106	0,00633776	0,00356074
64	0,003684000	0,00317471	0,00459375	0,00601618	0,00181254
65	0,007737000	0,00095052	0,00523479	0,00918888	0,00722797
66	0,006352000	0,00855806	0,00813884	0,00774304	0,00910802
67	0,009049333	0,00848414	0,00616266	0,00902497	0,00606798
68	0,001543333	0,00603407	0,00772965	0,00956085	0,00603407
69	0,007127333	0,00672806	0,00532262	0,00422198	0,00672806
70	0,008952000	0,00621492	0,00678481	0,00025618	0,00621492
71	0,012722333	0,00603837	0,00753425	0,01476461	0,00603837
72	0,007486667	0,00405657	0,00620225	0,00431144	0,00405657
73	0,003091333	0,00599851	0,00875124	0,00764032	0,00599851
74	0,003377000	0,00704068	0,00798568	0,00449493	0,00704068
75	0,001129333	0,01584553	0,00825951	0,01598061	0,01584553
76	0,051064000	0,01842220	0,00620225	0,00999287	0,01842220
77	0,002113000	0,00000000	0,00406641	0,01646671	0,00000000
78	0,000849000	0,01740602	0,00271323	0,02098139	0,01740602
79	0,036220000	0,01672888	0,01037899	0,01038759	0,01672888
80	0,030151000	0,01944242	0,01247981	0,03038352	0,01944242
81	0,046139000	0,00415217	0,00044793	0,04007492	0,00415217
82	0,040047000	0,00592784	0,01609203	0,02600597	0,00592784
83	0,014365333	0,00467673	0,01394188	0,02316863	0,00467673
84	0,048667333	0,02569368	0,01472128	0,02205825	0,02569368
85	0,058706333	0,01607870	0,02343534	0,03796344	0,01607870
86	0,056561000	0,00133855	0,02495649	0,02130482	0,00133855
87	0,048916333	0,00000000	0,01901352	0,01600686	0,00000000
88	0,067074000	0,01879645	0,02784056	0,00528997	0,01879645
89	0,074181000	0,02042700	0,02050916	0,01380004	0,02042700
90	0,114463667	0,03029394	0,02899366	0,06805154	0,03029394
91	0,072808667	0,01477702	0,02954398	0,03891019	0,01477702
92	0,045325667	0,01408988	0,03338529	0,05281075	0,01408988
93	0,096987000	0,00581538	0,03103553	0,01880065	0,00581538
94		0,02300350	0,04366441	0,03281350	0,02300350
95		0,01349167	0,00267292	0,05419382	0,01349167
96		0,02646739	0,05084060		0,02646739
97		0,02968467	0,03033241		0,02968467
98		0,02330225	0,02380940		0,02330225
99		0,00437060	0,01764397		0,00437060
100		0,01706837	0,02289729		0,01706837
101		0,03916314	0,01742993		0,03916314
102		0,02666044	0,01262058		0,02666044
103		0,02157759	0,00921602		0,02157759
104		0,01604701	0,00108657		0,01604701
105		0,01301883	0,00069193		0,01301883
106		0,05459675	0,00794080		0,05459675
107		0,04172041	0,05985485		0,04172041
108		0,06840649			0,06840649
109		0,04617142			0,04617142
110		0,04516420			0,04516420
111		0,03113823			0,03113823
112		0,07726497			0,07726497
113		0,05212841			0,05212841
114		0,00504018			0,00504018
115		0,03632880			0,03632880
116		0,06199983			0,06199983
117		0,03537346			0,03537346
118		0,01104695			0,01104695

119		0,06312488			0,06312488	
120		0,04252388			0,04252388	

A-Form	D1-68	D2-68	D3-68	A1	A2	A3
**chamber**	**lin. Vol. mm^3**	**lin. Vol. mm^3**	**lin. Vol. mm3**	**lin. Vol. mm^3**	**lin. Vol. mm^3**	**lin. Vol. mm^3**
3	0,00004926	0,00005343	0,00004167	0,00000233	0,00001100	0,00000333
4	0,00014562	0,00009845	0,00010692	0,00000333	0,00001733	0,00000000
5	0,00022522	0,00012566	0,00008241	0,00000033	0,00001767	0,00000667
6	0,00028909	0,00017470	0,00013729	0,00001067	0,00001133	0,00000400
7	0,00027076	0,00020661	0,00017062	0,00002667	0,00001467	0,00000433
8	0,00028731	0,00023839	0,00021464	0,00002533	0,00002233	0,00000667
9	0,00037580	0,00025657	0,00028594	0,00002133	0,00002000	0,00000667
10	0,00038107	0,00028223	0,00024742	0,00001367	0,00004633	0,00000733
11	0,00010133	0,00040022	0,00022594	0,00003133	0,00003067	0,00000633
12	0,00049086	0,00050894	0,00038563	0,00005600	0,00002100	0,00001300
13	0,00125263	0,00041519	0,00046366	0,00006433	0,00006467	0,00001567
14	0,00109848	0,00032595	0,00050335	0,00006667	0,00008700	0,00002133
15	0,00111413	0,00027525	0,00055972	0,00010333	0,00007833	0,00001200
16	0,00124380	0,00088826	0,00121030	0,00010800	0,00014433	0,00003933
17	0,00085012	0,00094014	0,00136666	0,00007000	0,00011567	0,00002833
18	0,00026305	0,00116944	0,00118042	0,00006533	0,00015700	0,00002600
19	0,00221380	0,00123771	0,00085491	0,00009800	0,00017433	0,00012600
20	0,00212100	0,00118892	0,00064157	0,00021533	0,00028833	0,00011267
21	0,00229540	0,00137920	0,00089417	0,00023733	0,00046300	0,00012700
22	0,00270363	0,00227971	0,00067682	0,00015733	0,00051433	0,00011367
23	0,00213065	0,00266824	0,00494101	0,00018300	0,00071100	0,00018733
24	0,00232033	0,00137030	0,00081213	0,00027300	0,00090067	0,00025000
25	0,00276408	0,00232003	0,00107301	0,00029433	0,00109667	0,00025400
26	0,00288366	0,00284566	0,00129363	0,00074067	0,00131333	0,00016000
27	0,00373475	0,00344884	0,00134487	0,00060600	0,00118600	0,00048967
28	0,00319730	0,00350790	0,00261999	0,00104567	0,00153033	0,00059133
29	0,00433785	0,00321973	0,00337909	0,00046667	0,00163500	0,00053700
30	0,00332579	0,00282160	0,00386887	0,00080133	0,00144767	0,00024867
31	0,00366613	0,00438490	0,00351175	0,00075967	0,00054967	0,00031000
32	0,00194378	0,00600923	0,00359410	0,00042033	0,00047400	0,00100600
33	0,00311311	0,00591054	0,00256554	0,00035733	0,00200200	0,00109033
34	0,00136887	0,00508331	0,00431277	0,00056033	0,00187500	0,00148233
35	0,00589143	0,00629079	0,00594525	0,00034167	0,00177167	0,00138200

36	0,00331948	0,00524175	0,00460192	0,00149367	0,00268633	0,00107367
37	0,00542817	0,00402803	0,00382868	0,00147167	0,00277733	0,00138200
38	0,00461417	0,00139083	0,00413443	0,00173767	0,00267333	0,00107367
39	0,00500496	0,00426246	0,00688745	0,00168133	0,00342167	0,00080933
40	0,00850285	0,00261426	0,00601748	0,00127967	0,00302767	0,00078700
41	0,00297380	0,00922352	0,00535535	0,00016000	0,00166000	0,00078867
42	0,01100404	0,01193084	0,00792854	0,00186800	0,00186533	0,00127067
43	0,01022238	0,01056840	0,00439259	0,00238367	0,00402467	0,00140367
44	0,01526659	0,00769287	0,00967010	0,00189033	0,00610433	0,00138167
45	0,01504263	0,00807986	0,00741655	0,00054033	0,00410233	0,00294867
46	0,01067237	0,02128132	0,00609229	0,00230633	0,00404300	0,00270933
47	0,01534678	0,01501056	0,01035149	0,00295667	0,00427400	0,00200700
48	0,00923154	0,02505926	0,00862215	0,00324300	0,00556067	0,00354767
49	0,00701536	0,02187429	0,00719296	0,00360400	0,00489167	
50	0,01336375	0,01859693	0,00017495	0,00182767	0,00583100	
51	0,01128377	0,01971227	0,01019950	0,00303367	0,00615867	
52	0,01265406	0,02244186		0,00327067	0,00160933	
53	0,01029204	0,02187188		0,00336733		
54	0,02509885			0,00665167		
55	0,02548348			0,00412033		
56	0,01611122			0,00630767		
57	0,03341462			0,00393167		
58	0,02627174			0,00251467		
59	0,00815116			0,00693633		
60				0,00730967		
61				0,00824500		
62				0,00623133		

A-Form	R1	R2	R3	R6
**chamber**	**lin. Vol. mm³**	**lin. Vol. mm³**	**lin. Vol. mm3**	**lin. Vol. mm³**
3	0,00002200	0,00001550	0,00002303	0,00001228
4	0,00003868	0,00002997	0,00000059	0,00001827
5	0,00006119	0,00004709	0,00003063	0,00003698
6	0,00007315	0,00002399	0,00008917	0,00003704
7	0,00005337	0,00002517	0,00012467	0,00005266
8	0,00009625	0,00012349	0,00014423	0,00004895
9	0,00010917	0,00013973	0,00017287	0,00005080
10	0,00016815	0,00018838	0,00014815	0,00007253
11	0,00008385	0,00022263	0,00021753	0,00011328
12	0,00014911	0,00011530	0,00014697	0,00019149
13	0,00019694	0,00018432	0,00023436	0,00009235
14	0,00025097	0,00028611	0,00031084	0,00008865
15	0,00028064	0,00020026	0,00031187	0,00018501
16	0,00028840	0,00042370	0,00030227	0,00027532
17	0,00037970	0,00045337	0,00048282	0,00015291
18	0,00044592	0,00014187	0,00069224	0,00030643
19	0,00056070	0,00035040	0,00041314	0,00039261
20	0,00078045	0,00066780	0,00041846	0,00021828
21	0,00097975	0,00066913	0,00056144	0,00047119
22	0,00213488	0,00118178	0,00122134	0,00048786
23	0,00076893	0,00113860	0,00101791	0,00077293
24	0,00130173	0,00236223	0,00127309	0,00137055
25	0,00122186		0,00101068	0,00102072
26	0,00080503	0,00119698	0,00152694	0,00053934
27	0,00077284	0,00147254	0,00153831	0,00086195
28	0,00285553	0,00063739	0,00118001	0,00106116

29	0,00248646	0,00082444	0,00235330	0,00132567
30	0,00499462	0,00114495	0,00088800	0,00104017
31	0,00348717	0,00126578	0,00203405	
32	0,00374811	0,00102396	0,00261984	0,00021797
33	0,00492243	0,00160245	0,00293090	0,00051836
34	0,00351463	0,00123552	0,00425249	0,00061861
35	0,00234378		0,00310761	0,00005834
36	0,00069349	0,00104160	0,00383152	0,00018803
37	0,00544696	0,00037609	0,00108833	0,00036211
38	0,00417786	0,00029976		0,00024229
39	0,00453896	0,00115978	0,00264871	0,00011612
40		0,00081462	0,00532775	0,00164211
41	0,00127471	0,00469109	0,00753556	0,00161902
42	0,00523009	0,00127944	0,00003735	0,00325069
43	0,00260183	0,00131671	0,00537868	
44	0,00531114	0,00107386	0,00225269	0,00163661
45	0,00356682	0,00030035	0,00132624	0,00436062
46	0,00305801	0,00057524	0,00213436	0,00332792
47	0,00207206	0,00025872	0,00026507	0,00233621
48	0,00058019	0,00012298	0,00003100	0,00069491
49	0,00200651	0,00719571	0,00072346	0,00576061
50	0,00144567	0,00486508	0,00102994	0,00333922
51		0,00382967	0,00052416	0,00508287
52	0,00073092		0,00518625	0,00039409
53	0,00037838	0,00867120		0,00408357
54	0,00129235	0,00168741	0,00589435	0,00578234
55	0,00421956		0,00190856	0,00409980
56	0,00279789		0,00525548	0,00385047
57	0,01356994		0,01041471	0,00916693
58	0,01049126		0,00835845	0,00452778
59	0,00465544		0,00299298	0,00841357
60	0,00364728		0,00080348	0,00297155
61	0,00177983			
62	0,00192214			
	0,00023517			
